# *In vivo* scAAV-based NeuroD1 gene therapy inhibiting glioblastoma growth

**DOI:** 10.1016/j.omton.2026.201324

**Published:** 2026-08-28

**Authors:** Debora Mazzetti, Pier Paolo Peruzzi, Himanshu Soni

**Affiliations:** 1 Harvey W. Cushing Neuro-oncology Laboratories (HCNL), Department of Neurosurgery, Harvard Medical School and Brigham and Women’s Hospital, Boston, MA, USA

## Main text

The poor prognosis of glioblastoma patients stems from glioblastoma’s characteristic behavior of rapid invasive growth, an immunologically cold microenvironment, tumor heterogeneity, and a strong tendency to recur, with a dismal median survival of 12–18 months.^1^ Among the many research strategies, which are being deployed to dismantle the aggressiveness of glioblastoma at pre-clinical stage is the potential to reprogram this glial tumor to neuronal-like state, which reduces its growth potential and subsequent invasive behavior while limiting off-target effects on neighboring tissue.^2^^,^^3^ In a recent issue of *Molecular Therapy Oncology*, the authors have taken the next step of delivering a neuronal re-programming transcription factor (TF) NeuroD1 to the tumor microenvironment *in vivo* using a self-complementary AAV (scAAV) platform.^4^ The findings highlight clinically relevant therapeutics to increase survival benefit in patients with glioblastoma.

The neuronal basic-helix-loop-helix (bHLH) superfamily of TFs is highly conserved throughout evolution for neuronal tissue development and maintenance. Among these are several TFs, such as NeuroD1, NeuroD2, NeuroD6, and Bhlhe22, which act as master regulators of post-mitotic neuronal differentiation.^5^ Alongside other neuronal reprogramming TFs such as ASCL1, NGN2, ZIC1, and SOX11, these TFs have emerged as powerful tools to redirect cellular fate. Overexpression of such TFs has been shown to derive the differentiation of glial cells into neurons.^6^ This approach has offered effective therapeutic benefits in pre-clinical models of neurodegenerative diseases, including ischemic stroke, Huntington disease, and Alzheimer disease.^6^ Among these TFs, NeuroD1 has emerged as one of the most potent therapeutic candidates in inducing maximum neuronal differentiation yield (∼90%) when delivered *in vivo* using a retroviral platform in a stab injury pre-clinical mouse model.^6^ Given the limited proliferative potential of neurons, several research groups have extended this reprogramming paradigm to brain tumors, successfully attenuating their growth potential with minimal side effects.^2^^,^^7^^,^^8^ NeuroD1 overexpression in multiple such studies has been shown to induce neuronal differentiation genes and electrophysiological activity, as well as reduce growth potential.^9^^,^^10^

Herein, Chen et al. present a clinically safe and translatable scAAV vector carrying the NeuroD1 gene and provide evidence of its potential to neuro-convert glioblastoma cells *in vitro* and *in vivo*. Having confirmed the glioma-specific infectivity of AAV serotype 6 and the enhanced GFP expression driven by the CMV promoter, the authors identified AAV6 with CMV-driven NeuroD1 expression as their clinical candidate (scAAV6-NeuroD1). scAAV6-NeuroD1 successfully attenuated proliferation and sphere formation in both mouse and human GBM cell lines *in vitro*, better than similar vector with AAV serotype 9. scAAV6-NeuroD1-infected GBM organoids showed enhanced expression of NeuN, loss of GFAP, and dismantled tissue depicting glioma-neuron conversion, and potential cytotoxic effect respectively, which the authors confirmed using TUNEL assay. Transcriptomic analysis of GBM organoids treated with scAAV6-NeuroD1 for 14 days confirmed a reduction in glioma-associated genes and an increase in neuronal genes associated with synaptogenesis and neuron maturation.

Triple intracranial administration of scAAV6-NeuroD1 in a U87-MG orthotopic cell-derived xenograft (CDX) mouse model, within 14 days of tumor implantation, resulted in an 81.5% reduction in tumor size, as confirmed by DAPI staining. NeuroD1 expression was detectable in tumors as early as 24 h post-injection, with increased NeuN and NeuroD1 co-staining observed 14 days post-injection, suggestive of glioma-to-neuron conversion. The authors also observed increased microglial infiltration, as assessed by Iba1 staining, 14 days post-injection; however, whether this infiltration reflects a pro-tumorigenic or anti-tumorigenic role remains unclear. It is also unclear what molecular mechanism mediated by NeuroD1 would cause such response. scAAV6-NeuroD1-treated mice with U87-MG showed delayed body weight loss and a significant 53% increase in median survival at a high-dose of 2 × 10^13^ vg/mL. Notably, combinatorial treatment with scAAV6-NeuroD1 and temozolomide (TMZ) conferred complete protection against tumor-associated lethality, underscoring a synergistic therapeutic interaction between gene-directed neuronal reprogramming and standard-of-care chemotherapy. Still, the molecular mechanism for this purported synergy is not explained.

Triple administration of scAAV6-NeuroD1 intracranial injections in immunocompetent orthotopic mouse GBM model GL261 also showed protection against tumor-associated weight loss, reduced tumor growth as depicted by tumor-associated luciferase intensity and enhancement of survival. However, the survival benefit remains inconclusive given the short 30-day endpoint window, which precludes definitive conclusions regarding whether scAAV6-NeuroD1 confers complete protection against lethality. Also, due to limitations in controls, the data do not provide evidence that the observed enhanced anti-tumor immunity is indeed mediated by the NeuroD1 delivery, vs. a simple result of the AAV infection. Furthermore, the authors also tested the efficacy of scAAV6-NeuroD1 in patient-derived xenograft (PDX) models. As expected, there was a dose-dependent reduction in proliferation potential of the PDX models *in vitro* and a significant attenuation of tumor growth in non-obese diabetic (NOD)/severe combined immunodeficiency (SCID) mouse models bearing subcutaneous (s.c.) PDX tumors.

Chen et al. further examined the clinical relevance of NeuroD1 across multiple glioma grades using several publicly available glioma datasets, including The Cancer Genome Atlas (TCGA) and Chinese Glioma Genome Atlas (CGGA), among others. NeuroD1 expression was found to correlate strongly with improved prognosis in glioma patients. Additionally, this analysis revealed a grade-dependent pattern of NeuroD1 expression, with IDH-mutant and other low-grade gliomas demonstrating significantly higher NeuroD1 transcript levels compared to high-grade gliomas. Collectively, these findings suggest that elevated NeuroD1 expression in gliomas is associated with a more favorable clinical outcome.

Taken together, Chen et al. present an important step forward for the field of cancer reprogramming therapy, in which a scAAV6 vector delivering NeuroD1 (scAAV6-NeuroD1) demonstrates consistent and statistically robust anti-tumor efficacy, although the majority of presented evidence comes from the U87 model, known to be particularly permissive to AAV infection and perhaps not the best GBM model available. The correlation of high NEUROD1 expression with improved prognosis across multiple independent patient cohorts (TCGA and CGGA) further strengthens the clinical relevance and biological plausibility of the approach. Nevertheless, the long-term persistence and immunomodulatory effects of scAAV6-NeuroD1 remain to be thoroughly characterized in orthotopic immunocompetent models. Equally important, extended safety evaluation is warranted to confirm the health and functional integrity of the induced neurons, a critical step in advancing this promising therapeutic candidate toward clinical application. Beyond these study-specific gaps, the AAV therapeutic approaches are accompanied with well-recognized challenges, such as the strong pre-existing or induced antibody-mediated neutralization of the AAVs triggering tumor recurrence. Also, strategies involving multiple infusions of AAVs to boost transduction efficiency risk high manufacturing and logistical costs while also provoking strong systemic toxicity. Addressing these translational hurdles will be essential to determine whether scAAV6-NeuroD1-mediated neuronal reprogramming can move from a compelling proof-of-concept into a viable therapeutic option for glioblastoma.

## Declaration of interests

The author declares no competing interests.
